# Establishment and Comparative Analysis of Four Mouse Models for the Study of Pancreatic Steatosis

**DOI:** 10.3390/ijms27104255

**Published:** 2026-05-10

**Authors:** Xinpeng Yin, Chenglin Hu, Chenxue Yin, Jiaying Li, Chengcheng Wang, Yupei Zhao

**Affiliations:** 1 Department of General Surgery, Peking Union Medical College Hospital, Peking Union Medical College, Chinese Academy of Medical Sciences, Beijing 100730, China; yinxinpeng0222@163.com (X.Y.);; 2 Key Laboratory of Research in Pancreatic Tumor, Chinese Academy of Medical Sciences, Beijing 100730, China; 3 State Key Laboratory of Complex, Severe, and Rare Diseases, Peking Union Medical College Hospital, Chinese Academy of Medical Science and Peking Union Medical College, Beijing 102602, China; 4 National Infrastructures for Translational Medicine, Peking Union Medical College Hospital, Beijing 100730, China

**Keywords:** pancreatic steatosis, mouse models, high-fat diet, *ob/ob* mice, caerulein, orthotopic injection, metabolic syndrome

## Abstract

Pancreatic steatosis is closely associated with metabolic disorders, pancreatitis, and pancreatic cancer, yet the underlying mechanisms remain incompletely understood and ideal animal models are lacking. We established and compared four mouse models based on distinct pathogenic mechanisms. In the high-fat diet (HFD)-fed model, pancreatic steatosis occurred later than hepatic steatosis. In *ob/ob* mice, despite severe obesity and hepatic steatosis, no significant intrapancreatic fat deposition was observed. In the caerulein combined with HFD model, caerulein markedly accelerated HFD-induced intrapancreatic fat accumulation, indicating a synergistic effect of inflammation and metabolic stress. Orthotopic injection of adipogenically differentiated 3T3-L1 cells established a focal model without altering systemic metabolism, enabling investigation of localized fat deposition. Collectively, these four models recapitulate metabolic, genetic, inflammatory, and localized mechanisms, exhibit divergent histological features, and provide a complementary platform for mechanistic studies and drug screening in pancreatic steatosis.

## 1. Introduction

In physiological conditions, a small amount of intrapancreatic fat is a normal component of the human pancreas, with deposition occurring through multiple pathways, including interlobular adipocyte aggregation, intracellular lipid droplets in acinar and islet cells, acinar-to-adipocyte transdifferentiation, and fat replacement of apoptotic acinar cells [1,2,3]. When excessive ectopic lipid deposition occurs within pancreatic parenchymal cells and the interstitium, it constitutes the pathological state of pancreatic steatosis, also termed nonalcoholic fatty pancreatic disease. Its pathogenesis is closely associated with obesity, insulin resistance, adipokine imbalance, and local chronic inflammation [4,5]. Imaging assessment, particularly chemical shift-encoded magnetic resonance imaging (MRI), is the preferred method for quantitative analysis of pancreatic fat in humans, allowing noninvasive evaluation of pancreatic fat content [6,7,8]. In animal studies, oil red O staining, hematoxylin-eosin (HE) staining, and immunohistochemistry (IHC) are commonly used to define lipid droplet distribution, inflammatory infiltration, and fibrosis [9]. Importantly, excessive intrapancreatic fat plays a significant role in the pathogenesis of type 2 diabetes; however, pancreatic steatosis is not an inevitable consequence of systemic obesity, suggesting the existence of local regulatory mechanisms independent of global metabolic status [1,10,11,12]. In the context of pancreatic diseases, excessive intrapancreatic fat accumulation plays a critical role in the transition from acute to chronic pancreatitis, and its progressive increase is considered an early sign of pancreatic cancer [13,14,15,16]. Moreover, the underlying causes of pancreatic steatosis encompass multiple factors, including metabolic syndrome, nonalcoholic fatty liver disease, alcohol consumption, and specific genetic disorders [17,18]. Notably, intrapancreatic fat is generally plastic, and its content can be reduced through various interventions such as low-calorie diets, exercise, bariatric surgery, and pharmacological treatments [19,20,21]. Although pancreatic steatosis has not yet been incorporated into the International Classification of Diseases (ICD) coding system and appears to lack direct clinical harm with a certain degree of reversibility [22], its presence serves as a crucial warning sign for the impending development of multiple diseases.

To investigate the pathological mechanisms and intervention strategies of pancreatic steatosis, several mouse models have been established. Among them, the high-fat diet (HFD)-fed model is the most commonly used, in which long-term HFD administration induces obesity, insulin resistance, and varying degrees of pancreatic fat deposition, thus reasonably mimicking metabolic syndrome-associated pancreatic steatosis; however, the extent of fat infiltration is substantially influenced by factors such as genetic background, diet composition, and feeding duration [13,23,24,25]. Nevertheless, an ideal mouse model that effectively recapitulates pancreatic steatosis remains lacking. Genetic obesity models, such as *ob/ob* mice (leptin-deficient), exhibit spontaneous severe obesity and hyperinsulinemia, with common pancreatic features including islet hyperplasia and inflammatory cell infiltration, and have been widely employed in obesity and type 2 diabetes research [26,27]. Although the HFD-fed, *ob/ob*, and caerulein + HFD models have been individually reported, none have been specifically established or systematically compared for the study of pancreatic steatosis. Previous reports focused primarily on obesity, diabetes, or pancreatitis, without dedicated characterization of intrapancreatic fat deposition and its associated pathological features (fibrosis, exocrine dysfunction, immune infiltration). To address this gap, we provide the first head-to-head comparison of these models specifically focusing on pancreatic steatosis and its related pathological alterations. Furthermore, we are the first to propose and validate a 3T3-L1 orthotopic injection model for focal pancreatic steatosis, which enables investigation of the causal relationship between localized fat deposition and pancreatic pathology independent of systemic metabolic disturbances. Based on this background, the present study established and systematically compared four mouse models of pancreatic steatosis based on distinct pathological mechanisms: the HFD-fed model, the *ob/ob* mouse model, the caerulein combined with HFD model, and the 3T3-L1 orthotopic injection model. By systematically comparing these models in terms of fat deposition degree and other parameters, this study aims to provide more comprehensive and reliable experimental tools for mechanistic investigation and drug screening in pancreatic steatosis, as well as to offer theoretical insights for clinical intervention across different etiological subtypes.

## 2. Results

### 2.1. Long-Term High-Fat Diet-Fed C57BL/6J Mouse Model Exhibits Pancreatic Steatosis

**Figure 1:**
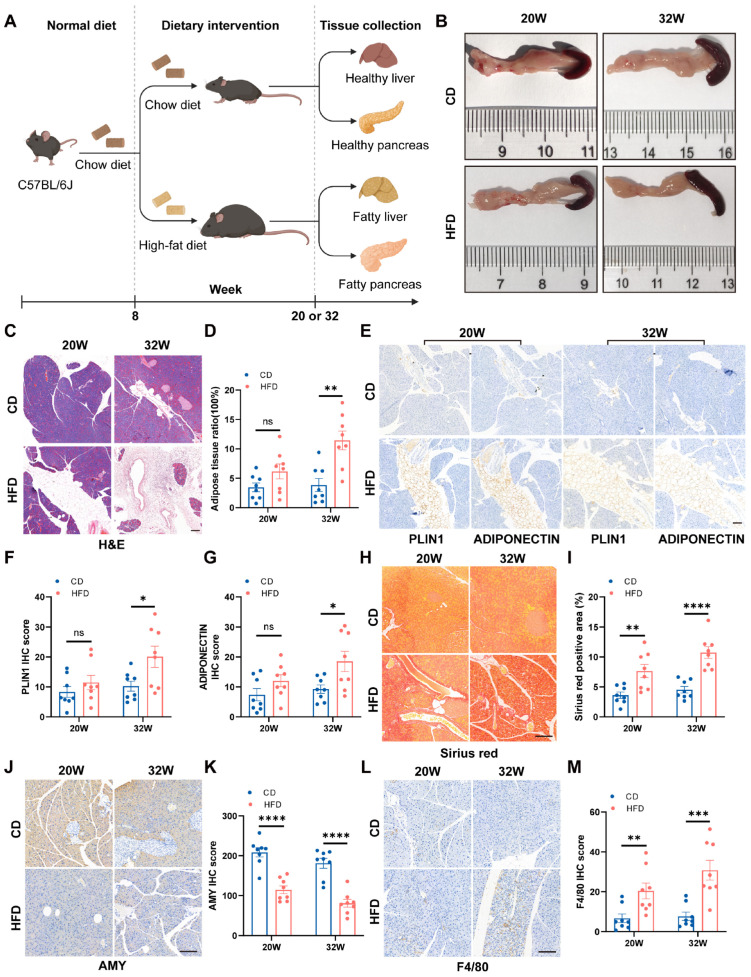
Long-term high-fat diet feeding induces pancreatic steatosis in C57BL/6J mice. (**A**) Schematic illustration of the HFD feeding regimen in C57BL/6J model, created on BioRender.com with permission for publication. (**B**) Representative gross images of the pancreas from CD and HFD-fed mice at 20 and 32 weeks of age. (**C**) Representative HE staining of pancreatic tissues at 20 and 32 weeks of age. (**D**) Quantification of adipose tissue ratio (area of intrapancreatic adipocytes/total area of the pancreatic section) by ImageJ software (n = 8). (**E**) Representative images of Perilipin1 and Adiponectin immunohistochemical staining of pancreatic sections from CD and HFD-fed mice at 20 and 32 weeks of age. (**F**,**G**) Semi-quantitative H-score analysis of Perilipin1 and Adiponectin staining. (**H**,**I**) Sirius red staining (**H**) and quantification (**I**) in CD and HFD-fed mice at 20 and 32 weeks (n = 8). (**J**,**K**) Amylase staining (**J**) and quantification (**K**) in CD and HFD-fed mice at 20 and 32 weeks (n = 8). (**L**,**M**) F4/80 immunohistochemistry (**L**) and quantification (**M**) show in CD and HFD-fed mice at 20 and 32 weeks (n = 8). Scale bars = 100 µm. Data are presented as mean ± SEM. Unpaired two-tailed Student’s *t*-test (**D**,**F**,**G**,**I**,**K**,**M**). * *p* < 0.05, ** *p* < 0.01, *** *p* < 0.001, **** *p* < 0.0001.

In this study, we first replicated and further explored the classic HFD-fed C57BL/6J mouse model. At 8 weeks of age, mice were randomly divided into two groups and fed either a chow diet (CD) or an HFD, respectively. Mice were euthanized at 20 weeks and 32 weeks of age, and liver and pancreatic tissues were collected (Figure 1A). Body weight was dynamically monitored throughout the experimental period. Significant differences in body weight between the two groups emerged after 6 weeks of HFD feeding and gradually stabilized after 12 weeks (Figure S1A). Gross anatomical examination of the pancreas revealed no notable differences in color, size, or morphology between the HFD-fed and chow-fed groups, except for a slightly yellowish appearance observed in a few individual pancreatic specimens from the 32-week HFD group (Figure 1B).

Liver and pancreatic tissues from C57BL/6J mice in both groups were collected at 20 and 32 weeks of age, processed for paraffin embedding, sectioning, and stained with HE. Histological examination revealed that at 20 weeks of age, the HFD-fed mice exhibited characteristic vacuolar lipid deposition within the cytoplasm of hepatocytes, with nuclei displaced to the periphery, indicating evident hepatic steatosis (Figure S1B). In the pancreas, although occasional small lipid droplets were observed within acinar cells, no statistically significant difference in intrapancreatic fat deposition was detected compared with the control group (Figure 1C,D). By 32 weeks of age, hepatic steatosis in the HFD-fed group had progressed markedly, with more extensive vacuolation and diffuse macrovesicular fatty changes in some regions (Figure S1B). Concurrently, substantial intrapancreatic fat deposition became apparent, characterized by widespread accumulation of lipid droplets both within acinar cells and in the interacinar spaces (Figure 1C,D). To further characterize the nature of intrapancreatic lipid deposition, we performed immunohistochemical staining for Perilipin1 and Adiponectin on paraffin-embedded pancreatic sections, followed by semi-quantitative H-score analysis using Image-J software. At 20 weeks of age, the HFD-fed group showed a trend toward increased expression of both adipocyte-related markers, but no statistically significant difference was observed compared with the control group. In contrast, at 32 weeks of age, the HFD-fed group exhibited markedly enhanced immunostaining intensity for both Perilipin1 and Adiponectin, and the H-scores were significantly higher than those of age-matched chow-fed controls (Figure 1E–G). These findings further confirm that prolonged HFD feeding induces deposition of mature and functionally active adipose tissue within the pancreatic parenchyma. 

Previous studies have reported that HFD feeding exacerbates pancreatic fibrosis in mice [28]. To investigate this in our model, we performed Sirius red staining on pancreatic sections. At 20 weeks of age, the HFD-fed group exhibited a higher degree of fibrosis compared with the CD group. This difference became even more pronounced at 32 weeks of age, indicating that prolonged HFD feeding progressively aggravates pancreatic fibrosis (Figure 1H,I). Additionally, we performed Amylase staining on mouse pancreatic sections. The results showed that the AMY staining intensity was markedly reduced in the HFD-fed group compared with the control group, indicating that long-term HFD feeding impairs pancreatic exocrine function (Figure 1J,K). These findings are consistent with previous reports. Studies have demonstrated that the HFD not only decreases the expression levels of digestive enzymes such as Amylase and Lipase in pancreatic tissue, but also impairs islet β-cell function, leading to reduced glucose-stimulated insulin secretion and glucose intolerance. Furthermore, HFD feeding induces insulin resistance and lipotoxicity, further exacerbating the disruption of both exocrine and endocrine pancreatic functions [29,30,31]. Finally, to investigate the effect of long-term high-fat feeding on immune cell infiltration within the pancreas, we performed F4/80 immunohistochemical staining. The results showed that the number of F4/80-positive macrophages was significantly increased in the HFD-fed group compared with the chow diet group, suggesting that prolonged HFD feeding may exacerbate local pancreatic inflammation by promoting macrophage recruitment (Figure 1L,M). These findings indicate that prolonged HFD feeding not only induces pancreatic steatosis in mice, with a notably later onset than hepatic steatosis and histological features distinct from those observed in the liver, but also promotes fibrosis, exocrine dysfunction, and immune cell infiltration in a time-dependent manner.

### 2.2. No Significant Fat Deposition Is Observed in the Pancreas of ob/ob Obese Mice

**Figure 2:**
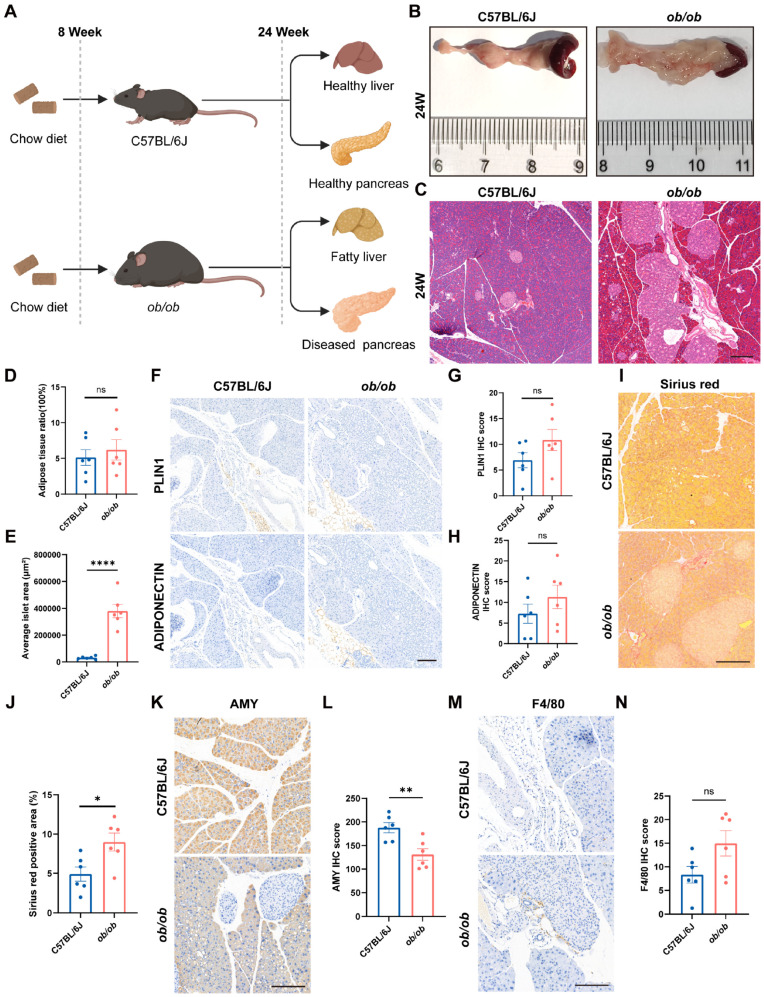
The *ob/ob* obese mouse model exhibits severe obesity and islet hyperplasia without significant intrapancreatic fat deposition. (**A**) Experimental design, created on BioRender.com with permission for publication. (**B**) Representative gross images of the pancreas from C57BL/6J and *ob/ob* mice at 24 weeks of age. (**C**) Representative HE staining of pancreatic tissues from C57BL/6J and *ob/ob* mice at 24 weeks of age. (**D**) Quantification of adipose tissue ratio by ImageJ software (n = 6). (**E**) Quantification of average islet area per mouse by ImageJ software (n = 6). (**F**–**H**) Representative images (**F**) and semi-quantitative H-score analysis of Perilipin1 (**G**) and Adiponectin (**H**) immunohistochemical staining of pancreatic sections from C57BL/6J and *ob/ob* mice at 24 weeks of age (n = 6). (**I**,**J**) Sirius red staining (**I**) and quantification (**J**) in C57BL/6J and *ob/ob* pancreas (n = 6). (**K**,**L**) Amylase staining (**K**) and quantification (**L**) in C57BL/6J and *ob/ob* mice (n = 6). (**M**,**N**) Representative images (**M**) and quantification (**N**) of F4/80 immunohistochemistry for macrophage infiltration in C57BL/6J and *ob/ob* mice (n = 6). Scale bars = 200 µm. Data are presented as mean ± SEM. Unpaired two-tailed Student’s *t*-test (**D**,**E**,**G**,**H**,**J**,**L**,**N**). * *p* < 0.05, ** *p* < 0.01, **** *p* < 0.0001.

Given that obesity is a well-established risk factor for pancreatic steatosis [32,33,34,35], the *ob/ob* obese mouse model was employed in this study. Mice were fed a CD from 8 weeks of age, and pancreatic fat deposition was compared with that of age-matched C57BL/6J mice at 24 weeks of age (Figure 2A). Body weight was dynamically monitored throughout the experimental period. Significant differences in body weight were observed between *ob/ob* and C57BL/6J mice, with the most pronounced differences occurring from 22 weeks of age onward (Figure S1C). Gross anatomical examination of the pancreas revealed that *ob/ob* mice exhibited enlarged pancreatic volume, with a more plump and thickened morphology and increased tissue firmness. Additionally, the surrounding adipose tissue was markedly increased and hypertrophied, with portions of the pancreas partially embedded within it (Figure 2B).

Subsequently, pancreatic and liver tissues from both groups were subjected to HE staining. The results revealed that, compared with chow-fed C57BL/6J mice, age-matched *ob/ob* mice exhibited diffuse lipid deposition in the liver, with hepatocytes extensively occupied by large macrovesicular lipid droplets, characteristic of macrovesicular steatosis (Figure S1D). This pattern differed from the microvesicular or mixed steatosis commonly observed in HFD-fed C57BL/6J mice (Figure S1B). In contrast, HE staining of the pancreas showed no significant difference in intrapancreatic fat deposition between the *ob/ob* and C57BL/6J groups (Figure 2C,D); however, islet volume was markedly increased in *ob/ob* mice (Figure 2E), consistent with previous reports [36,37,38]. To further characterize the pancreatic phenotype in *ob/ob* mice, we performed immunohistochemical staining for Perilipin1 and Adiponectin on paraffin-embedded pancreatic sections. A trend toward increased expression of both adipocyte-related markers was observed in the *ob/ob* group compared with controls, but no statistically significant difference was detected (Figure 2F,G). These results suggest that, despite severe systemic obesity and hepatic steatosis, the *ob/ob* pancreas does not develop substantial mature adipocyte deposition, consistent with the HE staining findings (Figure 2C,D).

We next assessed the degree of pancreatic fibrosis using Sirius red staining. The *ob/ob* group exhibited increased collagen deposition compared with C57BL/6J controls, indicating aggravated pancreatic fibrosis (Figure 2I,J). This finding is consistent with previous reports showing that obesity and hyperinsulinemia can promote fibrogenesis in various tissues, including the pancreas [31]. Notably, pancreatic stellate cells have been implicated as key mediators of obesity-associated pancreatic fibrosis through mechanisms involving oxidative stress and inflammatory signaling [39,40]. Furthermore, Amylase staining revealed markedly reduced enzymatic activity in the *ob/ob* group, indicating impaired pancreatic exocrine function (Figure 2K,L). Finally, to evaluate pancreatic immune cell infiltration, we performed F4/80 immunohistochemical staining. The *ob/ob* group showed a trend toward increased numbers of F4/80-positive macrophages compared with controls, but no statistically significant difference was observed (Figure 2M,N). Collectively, although *ob/ob* mice display severe obesity, hepatic steatosis, and islet hyperplasia, they do not develop substantial intrapancreatic fat deposition or mature adipocyte accumulation. However, pancreatic fibrosis and exocrine dysfunction are evident, while macrophage infiltration remains unchanged, suggesting that this mouse model requires further optimization for the study of pancreatic steatosis.

### 2.3. Caerulein Combined with High-Fat Feeding Induces Pancreatic Steatosis in Mice

**Figure 3:**
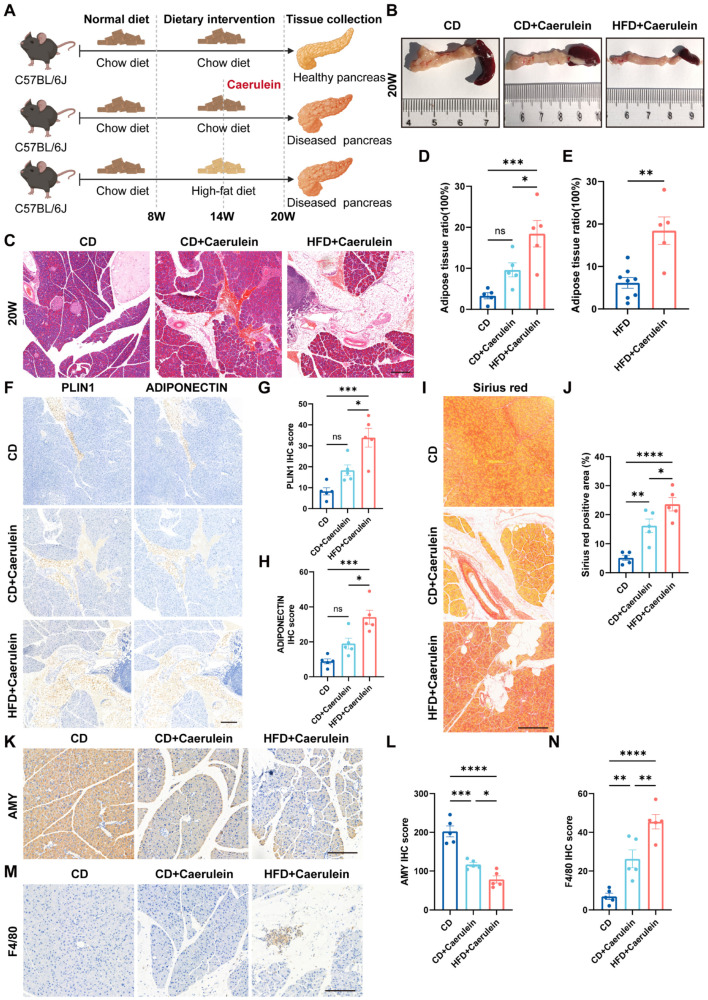
Synergistic induction of pancreatic steatosis by caerulein and high-fat feeding in mice. (**A**) Schematic overview of caerulein and dietary interventions, created on BioRender.com with permission for publication. (**B**) Representative gross images of the pancreas from the CD, CD + caerulein, and HFD + caerulein groups. (**C**) Representative HE staining of pancreatic tissues from the CD, CD + caerulein, and HFD + caerulein groups. (**D**) Quantification of adipose tissue ratio by ImageJ software (n = 5). (**E**) Comparison of pancreatic steatosis severity between the HFD + caerulein group and the HFD-alone group from Figure 1 (HFD n = 8, HFD + caerulein n = 5). (**F**–**H**) Perilipin1 (**F**) and Adiponectin (**G**) immunohistochemical staining and H-score analysis (**H**) of three groups (n = 5). (**I**,**J**) Sirius red staining (**I**) and quantification (**J**) of three groups (n = 5). (**K**,**L**) Amylase staining (**K**) and quantification (**L**) of three groups (n = 5). (**M**,**N**) F4/80 immunohistochemical staining (**M**) and quantification (**N**) of three groups (n = 5). Scale bars = 200 µm. Data are presented as mean ± SEM. One-way ANOVA with Tukey’s post hoc test (**D**,**G**,**H**,**J**,**L**,**N**), unpaired two-tailed Student’s *t*-test (**E**). * *p* < 0.05, ** *p* < 0.01, *** *p* < 0.001, **** *p* < 0.0001.

Based on the established association between pancreatic steatosis and chronic pancreatitis [10,17], caerulein was administered to C57BL/6J mice to establish a chronic pancreatitis model, combined with HFD feeding for 12 weeks. Mice were euthanized at 20 weeks of age for subsequent experiments (Figure 3A). Body weight was dynamically monitored throughout the experimental period. No significant difference in body weight was observed between the CD group and the CD plus caerulein (CD + caerulein) group; however, mice in the HFD plus caerulein (HFD + caerulein) group exhibited a marked increase in body weight (Figure S1E). Macroscopic inspection of the pancreas in both the CD + caerulein and HFD + caerulein groups revealed pronounced atrophy, with reduced size and thickness and increased tissue firmness (Figure 3B), consistent with previous reports [41,42].

HE staining revealed that, compared with the CD group, the CD + caerulein group exhibited no statistically significant difference in intrapancreatic fat deposition but showed a trend toward increase. In contrast, the HFD + caerulein group displayed a marked elevation in intrapancreatic fat deposition. Moreover, the HFD + caerulein group also exhibited significantly higher intrapancreatic fat deposition compared with the CD + caerulein group (Figure 3C,D). When the HFD + caerulein group, which underwent 12 weeks of HFD feeding, was compared with the HFD-alone group from Figure 1, the degree of pancreatic steatosis was substantially greater in the HFD + caerulein group (Figure 3E). To further evaluate the deposition of mature adipose tissue in the pancreas, we performed immunohistochemical staining for Perilipin1 and Adiponectin on paraffin-embedded pancreatic sections from the three groups, followed by semi-quantitative H-score analysis. Compared with the CD group, the CD + caerulein group showed a trend toward increased expression of both adipocyte-related markers, but no statistically significant difference was observed. In contrast, the HFD + caerulein group exhibited markedly enhanced staining intensity, with H-scores significantly higher than those of the CD group. Moreover, compared with the CD + caerulein group, the HFD + caerulein group also showed significantly increased expression of Perilipin1 and Adiponectin (Figure 3F–H). These findings indicate that the combination of caerulein-induced inflammation and high-fat feeding synergistically promotes the deposition of mature and functionally active adipose tissue within the pancreatic parenchyma, further supporting the synergistic role of inflammation and metabolic stress in pancreatic steatosis.

Then, we assessed the degree of pancreatic fibrosis by Sirius red staining. Compared with the CD group, the CD + caerulein group exhibited markedly increased collagen deposition, while the HFD + caerulein group showed even more severe fibrosis than the CD + caerulein group, indicating extensive collagen accumulation (Figure 3I,J). Furthermore, Amylase staining revealed that exocrine function was impaired in the CD + caerulein group and further deteriorated in the HFD + caerulein group (Figure 3K,L). These findings are consistent with previous reports demonstrating that caerulein-induced chronic inflammation disrupts pancreatic exocrine function and promotes fibrosis, with high-fat diet exacerbating these detrimental effects [43,44]. Finally, we evaluated macrophage infiltration by F4/80 immunohistochemical staining. The results showed a significant increase in F4/80-positive macrophages in the CD + caerulein group compared with the CD group, and the HFD + caerulein group exhibited an even greater extent of macrophage infiltration (Figure 3M,N). This observation is consistent with previous reports linking caerulein-induced pancreatitis to enhanced macrophage recruitment [45,46,47]. These findings suggest that caerulein treatment alone has a limited effect on inducing pancreatic steatosis in C57BL/6J mice, yet it markedly accelerates the progression and extent of intrapancreatic fat deposition, mature adipocyte accumulation, fibrosis, exocrine dysfunction, and macrophage infiltration in mice fed a HFD, demonstrating a synergistic effect of inflammation and metabolic stress in promoting pancreatic steatosis and associated pathology.

### 2.4. A Mouse Model of Localized Pancreatic Fat Deposition Induced by Orthotopic Injection of 3T3-L1 Cells

**Figure 4:**
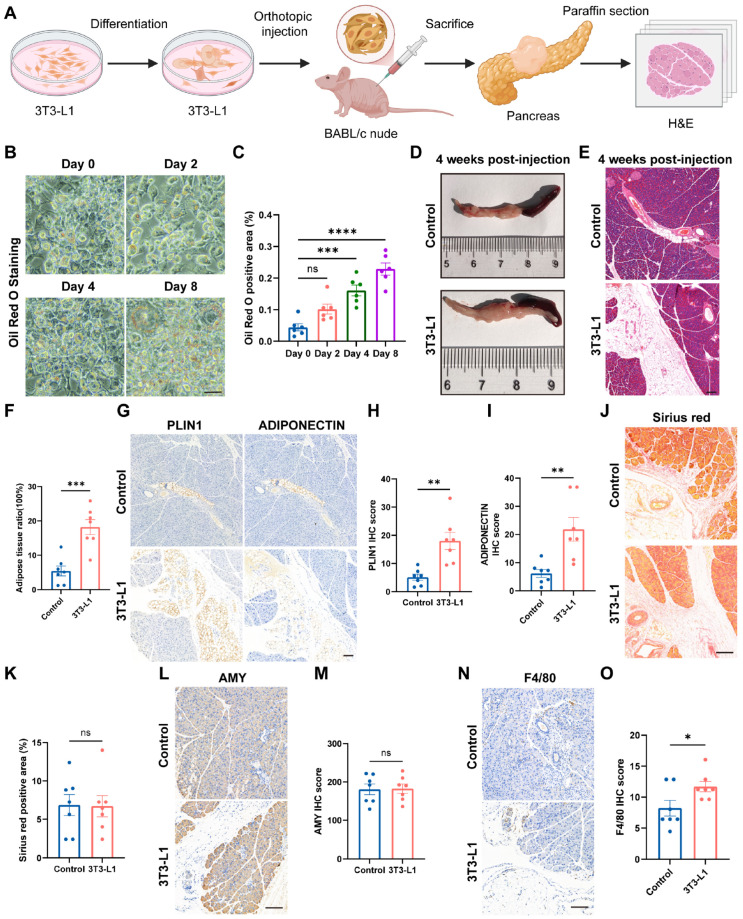
Orthotopic injection of 3T3-L1 cells induces localized pancreatic fat deposition in mice. (**A**) Study design of orthotopic 3T3-L1 injection, created on BioRender.com with permission for publication. (**B**) Oil red O staining of 3T3-L1 cells at indicated time points during adipogenic differentiation. (**C**) Quantification of Oil red O positive area by ImageJ software (n = 6). (**D**) Representative gross images of the pancreas at 4 weeks post-injection. (**E**) Representative HE staining of pancreatic tissues from control and 3T3-L1 injection groups. (**F**) Quantification of adipose tissue ratio by ImageJ software (n = 7). (**G**–**I**) Representative images of Perilipin1 (**G**) and Adiponectin (**H**) immunohistochemical staining and H-score analysis (**I**) of two groups (n = 7). (**J**,**K**) Sirius red staining (**J**) and quantification (**K**) of collagen deposition between the two groups (n = 7). (**L**,**M**) Amylase staining (**L**) and quantification (**M**) between the two groups (n = 7). (**N**,**O**) F4/80 immunohistochemical staining (**N**) and quantification (**O**) between the two groups (n = 7). Scale bars = 100 µm. Data are presented as mean ± SEM. One-way ANOVA with Tukey’s post hoc test (**C**), unpaired two-tailed Student’s *t*-test (**F**,**H**,**I**,**K**,**M**,**O**). * *p* < 0.05, ** *p* < 0.01, *** *p* < 0.001, **** *p* < 0.0001.

The three previously described mouse models primarily influence diffuse intrapancreatic fat deposition by altering systemic metabolic status. To establish a focal model of pancreatic steatosis, 3T3-L1 cells were induced toward adipogenic differentiation in vitro and subsequently injected orthotopically into the pancreas of BABL/c nude mice (Figure 4A). In vitro adipogenic differentiation of 3T3-L1 cells was first performed, and oil red O staining revealed a gradual increase in lipid droplet content over the induction period (0, 2, 4, and 8 days) (Figure 4B,C). As previously reported, optimal adipogenic capacity upon subcutaneous transplantation is achieved following approximately one week of in vitro adipogenic preconditioning [48]. Accordingly, 3T3-L1 cells induced for 8 days were selected for orthotopic pancreatic injection into BABL/c nude mice, with a follow-up period of 4 weeks. No significant difference in body weight was observed between the two groups during this period (Figure S1F). Four weeks post-injection, pancreatic tissues were collected for gross observation and HE staining. Compared with the control group, the 3T3-L1 injection group exhibited visible focal protrusions in the pancreatic body or tail (Figure 4D). HE staining further demonstrated a significantly increased pancreatic fat fraction in the 3T3-L1 injection group (Figure 4E,F). To further characterize the maturity of focal pancreatic fat deposition, we performed immunohistochemical staining for Perilipin1 and Adiponectin on paraffin-embedded pancreatic sections from both groups (Figure 4G). Compared with the control group, the 3T3-L1 orthotopic injection group exhibited markedly increased staining intensity for both adipocyte-related markers, indicating the formation of mature and functionally active adipose tissue at the injection site (Figure 4H,I). This finding is consistent with the increased pancreatic fat fraction observed by HE staining and further validates that this model successfully recapitulates focal mature fat deposition in the pancreas. 

We further assessed the degree of pancreatic fibrosis by Sirius red staining. The results showed no significant difference in collagen deposition between the 3T3-L1 orthotopic injection group and the control group (Figure 4J,K). Similarly, Amylase staining revealed no significant difference in pancreatic exocrine function between the two groups (Figure 4L,M). These findings indicate that this focal model did not induce apparent pancreatic fibrosis or exocrine dysfunction within 4 weeks post-injection, suggesting that this model can be specifically used to study the direct effects of localized pancreatic fat deposition on the pancreas, without confounding factors such as fibrosis or exocrine dysfunction. Finally, we evaluated macrophage infiltration in the pancreas by F4/80 immunohistochemical staining. The results showed a mild increase in the number of F4/80-positive macrophages in the 3T3-L1 orthotopic injection group compared with the control group (Figure 4N,O). This observation suggests that focal fat deposition alone can induce a certain degree of local inflammatory response, even in the absence of systemic metabolic disturbances. These findings indicate that orthotopic injection of adipogenically differentiated 3T3-L1 cells into the pancreas of BABL/c nude mice establishes a reliable focal pancreatic steatosis model, characterized by mature adipocyte deposition without significant fibrosis or exocrine dysfunction, and with only mild local macrophage infiltration, thereby providing a valuable tool to investigate the causal relationship between localized pancreatic fat deposition and pancreatic pathology independent of systemic metabolic disturbances.

## 3. Discussion

In this study, we systematically characterized four distinct mouse models of pancreatic steatosis, each representing a different pathogenic mechanism. Long-term HFD feeding in C57BL/6J mice successfully induced pancreatic fat deposition, albeit with a later onset than hepatic steatosis, and the histological features of pancreatic fat differed from those observed in the liver. In contrast, despite exhibiting severe obesity and pronounced hepatic steatosis, *ob/ob* mice did not develop substantial intrapancreatic fat deposition, indicating that systemic obesity alone is insufficient to drive pancreatic steatosis in this genetic background. When chronic pancreatitis was induced by caerulein in combination with HFD feeding, we observed a marked acceleration of pancreatic fat accumulation compared with HFD alone, highlighting the synergistic effect of inflammation and metabolic stress. Finally, orthotopic injection of adipogenically differentiated 3T3-L1 cells into the pancreas established a focal model of pancreatic steatosis that effectively excluded systemic metabolic confounders, enabling investigation of localized fat deposition.

In the present study, we systematically re-evaluated the classic HFD-fed C57BL/6J mouse model and found that the onset of pancreatic steatosis occurred significantly later than that of hepatic steatosis—the liver exhibited marked vacuolar lipid deposition at 20 weeks of age, whereas substantial intrapancreatic fat deposition did not appear until 32 weeks. This temporal disparity suggests that the pancreas may be more resistant to ectopic lipid deposition than the liver. Notably, prolonged HFD feeding not only induced pancreatic steatosis but also promoted fibrosis, exocrine dysfunction, and macrophage infiltration in a time-dependent manner. This temporal disparity suggests that the pancreas may be more resistant to ectopic lipid deposition than the liver, potentially attributable to differences in the expression profiles of lipid metabolism-related enzymes, local fatty acid uptake capacity, and the regulation of lipid droplet-coating proteins [49,50]. Of note, pancreatic tissue exhibits adaptive responses to lipid loading that differ from those of the liver, likely reflecting inherent differences between pancreatic acinar cells and hepatocytes in fatty acid oxidation capacity, lipid droplet formation, and lipid export mechanisms [51]. Moreover, HFD-induced pancreatic steatosis is characterized by a mixture of microvesicular and macrovesicular lipid droplets, contrasting with the predominantly macrovesicular steatosis observed in the liver, further highlighting the organ-specific heterogeneity in metabolic responses to lipid challenge [52]. Accumulating evidence indicates that the development of pancreatic steatosis is not solely dependent on systemic obesity but is tightly regulated by local lipid metabolic networks [25,53]. This model reasonably recapitulates the natural progression of metabolic syndrome-associated pancreatic steatosis; however, given its prolonged induction period (exceeding 24 weeks) and susceptibility to factors such as genetic background and dietary composition, particularly fatty acid profile [54], careful standardization of experimental duration and conditions is essential to ensure comparability and reproducibility of findings.

Despite exhibiting severe obesity, hyperinsulinemia, and typical macrovesicular hepatic steatosis, *ob/ob* mice do not develop substantial intrapancreatic fat deposition but instead show a marked increase in islet volume [55,56]. This observation aligns with clinical findings that not all obese individuals develop pancreatic steatosis, suggesting that the susceptibility of the pancreas to lipid deposition is regulated by mechanisms independent of systemic obesity [57]. Notably, although *ob/ob* mice lacked significant pancreatic fat deposition, pancreatic fibrosis and exocrine dysfunction were still evident, while macrophage infiltration remained unchanged. Moreover, recent studies have revealed significant differences in fat distribution, hepatic inflammation, and insulin secretion between *ob/ob* and *db/db* mice, despite both models displaying severe obesity, further indicating that the absence of leptin signaling exerts heterogeneous metabolic effects across different organs [58,59]. Due to the complete deficiency of leptin signaling in *ob/ob* mice, their energy metabolism and inflammatory status differ fundamentally from human polygenic obesity. As reviewed in Bentham Science, although these monogenic mutation models recapitulate certain phenotypes of obesity and type 2 diabetes, they do not reflect the true etiology of human disease, and researchers must fully acknowledge their limitations [60]. This may explain why this model fails to develop significant pancreatic steatosis. Furthermore, the pronounced islet hyperplasia in *ob/ob* mice may inhibit acinar cell lipid accumulation through local insulin signaling activation. Leptin is known to directly act on pancreatic β-cells to inhibit insulin secretion; *ob/ob* mice lack this negative feedback regulation, resulting in severe hyperinsulinemia [61,62,63]. Although this compensatory islet hyperplasia maintains glucose homeostasis, it may influence acinar cell lipid metabolism through paracrine mechanisms. Therefore, while this model remains suitable for obesity and diabetes research, its utility for mechanistic studies of pancreatic steatosis is limited, suggesting that future investigations should carefully consider genetic backgrounds or combine this model with other interventions, such as high-fat diet feeding, to induce pancreatic phenotypes.

We further provided the first systematic comparison of caerulein-induced chronic pancreatitis combined with HFD feeding in the context of pancreatic steatosis. Caerulein treatment alone had a limited effect on pancreatic fat accumulation, but when combined with HFD, it markedly accelerated intrapancreatic fat deposition, mature adipocyte accumulation, fibrosis, exocrine dysfunction, and macrophage infiltration compared with HFD alone, clearly demonstrating a synergistic effect of inflammation and metabolic stress. This “two-hit” model effectively recapitulates the clinical observation that patients with chronic pancreatitis frequently exhibit concomitant pancreatic steatosis [11,64]. The underlying mechanisms may involve multiple pathways. First, the release of pro-inflammatory cytokines such as IL-6 and TNF-α in the inflamed pancreas can activate the NF-κB pathway and upregulate adipogenic transcription factors including PPARγ and SREBP-1c, thereby promoting lipid synthesis and accumulation [65,66,67,68]. Second, inflammation-associated metabolic stress may impair mitochondrial function in acinar cells, leading to defective fatty acid oxidation and subsequent ectopic lipid deposition [13,69]. Additionally, local pancreatic fibrosis may alter the tissue microenvironment and activate pancreatic stellate cells, potentially further facilitating adipocyte infiltration and differentiation [70]. Notably, recent evidence indicates that inflammation in the exocrine pancreas can directly impair insulin secretion, even without a reduction in β-cell mass [71,72]. Moreover, the decrease in pancreatic amylase immunoreactivity after chronic caerulein treatment is explained by supramaximal stimulation-induced zymogen depletion. Prolonged caerulein exposure uncouples enzyme synthesis from secretion, leading to exhaustion of intracellular amylase stores. This is a well-documented feature of caerulein-induced chronic pancreatitis, where reduced tissue amylase reflects acinar cell dysfunction and exocrine insufficiency rather than preserved function [73,74,75]. This model thus provides a powerful tool for investigating the synergistic mechanisms of inflammatory and metabolic interactions in pancreatic steatosis and is particularly suitable for exploring lipid metabolic remodeling during the pathological progression of acute and chronic pancreatitis.

In contrast to the three diffuse models described above, we successfully established a focal pancreatic steatosis model by orthotopically injecting adipogenically differentiated 3T3-L1 cells into the pancreas. This model is not accompanied by weight gain or systemic lipid metabolic disturbances. The injected cells formed mature adipose tissue at the injection site (Perilipin1/Adiponectin double-positive), without inducing significant pancreatic fibrosis or exocrine dysfunction. However, F4/80 immunohistochemical staining revealed mild macrophage aggregation in the focal fat deposition area, suggesting that even in the absence of systemic metabolic disturbances, localized adipose tissue itself can induce a certain degree of local inflammatory response, likely through the secretion of chemokines such as CCL2 [46]. Accumulating evidence indicates that pancreatic steatosis is closely associated with the development and progression of pancreatitis, fibrosis, and pancreatic cancer [21,76,77]; thus, this focal model provides a valuable experimental tool for dissecting the causal relationships among these conditions, particularly for addressing the critical question of whether pancreatic fat serves as a causative factor or merely an epiphenomenon. Nevertheless, this model has certain limitations. The exogenous adipocytes differ from endogenous adipocytes in origin and local microenvironment, and both the injection procedure itself and the Matrigel vehicle may elicit a foreign body response or mild sterile inflammation. Therefore, rigorous sham-operated controls are essential when utilizing this model to exclude potential confounding effects related to the surgical procedure and the vehicle alone.

In summary, this study establishes a panel of four mouse models that collectively recapitulate metabolic, genetic, inflammatory, and focal pathogenic mechanisms of pancreatic steatosis. To our knowledge, this work provides the first head-to-head comparison of these existing models specifically focusing on intrapancreatic fat deposition and its associated pathological features (fibrosis, exocrine dysfunction, and immune infiltration). Moreover, we are the first to propose and validate the 3T3-L1 orthotopic injection model for focal pancreatic steatosis, which enables investigation of the causal relationship between localized fat deposition and pancreatic pathology independent of systemic metabolic disturbances. The systematic integration of these four mechanistically distinct models within a single study is itself unprecedented and offers a multidimensional experimental toolkit for future research. This side-by-side comparison allows investigators to select the most appropriate model for their specific questions, and provides a foundation for mechanistic studies—for example, using gene knockout in the HFD model to interrogate lipid metabolic genes such as PPARγ and SREBP-1c, applying inflammatory pathway inhibitors in the caerulein + HFD model to dissect crosstalk between inflammation and lipid accumulation, or co-injecting cytokines/drugs in the focal model to study local interactions. Integration with multi-omics technologies (single-cell transcriptomics, spatial transcriptomics, lipidomics) will further elucidate heterogeneity and common molecular mechanisms across etiological contexts. Collectively, this work provides a novel and versatile platform for mechanistic investigation and therapeutic development in pancreatic steatosis.

Several limitations should be acknowledged. First, all models are based on mice, and inherent species differences in pancreatic anatomy, metabolic regulation, and immune microenvironment necessitate validation of the findings in human clinical samples. Second, although the HFD model recapitulates key features of metabolic syndrome-associated pancreatic steatosis, its outcomes are influenced by dietary composition, particularly fatty acid profile, as well as feeding duration, which may lead to variability across laboratories; thus, rigorous standardization of experimental conditions is essential. The *ob/ob* model, due to complete deficiency of leptin signaling, exhibits profound differences in energy metabolism and inflammatory status compared with human polygenic obesity, limiting its ability to fully recapitulate human obesity-related pancreatic steatosis. In the focal pancreatic steatosis model, precise control of cell number and injection site is critical, and the immunodeficient nature of nude mice restricts its application in immune-related studies. Future efforts may explore alternative construction strategies in immunocompetent mice.

## 4. Materials and Methods

### 4.1. Animals

Animal feeding environment and animal experiments in this study were performed according to the guidelines of the Institutional Animal Care and Use Committee (Beijing, China, XHDW-2024-52). C57BL/6J and BALB/c nude (aged 8 weeks) mice were obtained from Beijing HFK Bioscience. Heterozygous *ob/+* mice were obtained from the Jackson Laboratory, and homozygous *ob/ob* mice were generated by intercrossing these heterozygotes. All animals were housed in the animal facility at Peking Union Medical College Hospital (PUMCH), with ad libitum access to drinking water and a standard CD (10% of calories from fat). All procedures were conducted in accordance with institutional guidelines and were approved by the institutional animal care and use committee of the PUMCH.

### 4.2. Experimental Design

For the long-term HFD (60% kcal fat, Research Diets, New Brunswick, NJ, USA) feeding model, 8-week-old C57BL/6J mice were randomly assigned to two groups: one group continued to receive a standard CD (HFK Bioscience, Beijing, China), while the other group was fed an HFD. Mice were euthanized at 20 and 32 weeks of age, and pancreatic and liver tissues were collected, processed for paraffin embedding, and sectioned. Body weight was measured every two weeks throughout the experimental period.

For the *ob/ob* obese mouse model, *ob/ob* mice and age-matched (8-week-old) C57BL/6J mice were fed a CD. Mice were euthanized at 24 weeks of age, and pancreatic and liver tissues were collected, processed for paraffin embedding, and sectioned. Body weight was measured every two weeks throughout the experimental period.

For the combined caerulein and HFD intervention model, 8-week-old C57BL/6J mice were randomly divided into three groups. The first group was maintained on a CD throughout the experiment as a control. The second group was also fed a CD and subjected to caerulein induction starting at 14 weeks of age. The third group was fed a HFD and subjected to caerulein induction starting at 14 weeks of age. All mice were euthanized at 20 weeks of age, and pancreatic tissues were collected, processed for paraffin embedding, and sectioned. Body weight was measured every two weeks throughout the experimental period. Caerulein induction was performed by intraperitoneal injection of caerulein (50 μg/kg body weight, Sigma-Aldrich, St. Louis, MO, USA) six times per day, three days per week, for six consecutive weeks, as previously described. Mice were sacrificed five days after the last injection [78].

All mice in the HFD-alone group (from mouse model 1) and the CD or HFD + caerulein group (from mouse model 3) were housed, fed, and processed in parallel.

For the 3T3-L1 orthotopic pancreatic transplantation model, 3T3-L1 cells (Cell Resource Center of Peking Union Medical College, Beijing, China) were first induced to differentiate under adipogenic conditions in vitro for approximately one week. Subsequently, 1 × 10^6^ cells per mouse were injected orthotopically into the body of the pancreas of BALB/c nude mice. Control mice received an equal volume and concentration of Matrigel (Corning, Bedford, MA, USA) without cells. Mice were euthanized 4 weeks post-transplantation, and pancreatic tissues were collected, processed for paraffin embedding, and sectioned. Body weight was measured weekly throughout the experimental period.

### 4.3. Tissue Collection

Mice were anesthetized with isoflurane, placed in a supine position, and the abdominal cavity was opened via a midline incision. The inferior vena cava or heart was cannulated and perfused with physiological saline until the liver turned pale to remove circulating blood. The liver was carefully dissected on ice, and the left lateral lobe, right lobe, and caudate lobe were completely removed and fixed in pre-chilled 4% paraformaldehyde. For pancreatic tissue collection, the tail of the pancreas was localized along the spleen, and peripancreatic fat and connective tissue were gently dissected along the pancreatic duct and splenic vessels. The pancreatic tail, body, and head were sequentially excised with minimal traction. The pancreas was immediately placed in 4% paraformaldehyde for fixation. Both liver and pancreatic tissues were fixed for 24–48 h, then transferred to 70% ethanol for storage prior to paraffin embedding and sectioning. All procedures were performed rapidly on ice to preserve tissue morphology and structural integrity.

### 4.4. H&E Staining

For histological examination, harvested tissues were fixed in 4% paraformaldehyde in phosphate-buffered saline (PBS) for 24–48 h. Following fixation, samples were dehydrated through a graded ethanol series, cleared in xylene, and embedded in paraffin blocks. Sections were cut at a thickness of 5 μm, mounted onto glass slides, and dried. After deparaffinization and rehydration, sections were stained with H&E according to standard protocols. Briefly, sections were immersed in hematoxylin for 5 min, rinsed, differentiated in acid alcohol, blued in lithium carbonate solution, and then counterstained with eosin for 1–2 min. Stained sections were dehydrated, cleared, and mounted with neutral resin for microscopic examination. 

Quantification of adipose tissue ratio and average islet area was performed using ImageJ software (Version 1.54, https://imagej.net/ij/, NIH, Bethesda, MD, USA, accessed on 24 April 2026). Adipose tissue ratio was defined as the area of intrapancreatic adipocytes divided by the total area of the pancreatic section. Average islet area was calculated as the mean islet area per mouse. For both the control and H&E-stained experimental groups, 5–10 images per animal were evaluated.

### 4.5. Immunohistochemistry Analysis

For IHC analysis, the specific procedure was performed as previously described. Briefly, paraffin-embedded pancreatic sections were deparaffinized, rehydrated, and subjected to antigen retrieval. Endogenous peroxidase activity was blocked, and sections were incubated with primary antibodies overnight at 4 °C. The primary antibodies and dilutions used were as follows: anti-Adiponectin (1:200, ab22554, Abcam, Cambridge, UK), anti-PLIN1 (1:100, 9349, Cell Signaling Technology, Danvers, MA, USA), anti-Amylase (1:1600, 3796, Cell Signaling Technology), and anti-F4/80 (1:200, 70076, Cell Signaling Technology). Following incubation with appropriate HRP-conjugated secondary antibodies, signals were visualized using diaminobenzidine (DAB) and counterstained with hematoxylin. Images were randomly acquired using a digital pathology device (3DHISTECH, Budapest, Hungary). Semi-quantitative assessment of staining intensity and area was performed using ImageJ software (version 1.54) based on an immunoreactive score system.

### 4.6. Sirius Red Staining

The Sirius Red Staining Kit (C0190S) was purchased from Beyotime, and the staining procedure was performed according to the manufacturer’s protocol. Briefly, paraffin sections were dewaxed in xylene (replaced with fresh xylene and repeated once), then rehydrated through graded ethanol (absolute, 90%, 80%, and 70%) and washed with distilled water. Tissue areas were outlined with an immunohistochemistry pen, and hematoxylin staining solution was applied for 10 min, followed by rinsing, differentiation in acid-alcohol, and blueing under running tap water. Subsequently, Sirius Red staining solution was added for 10–15 min (5–10 min for easily stained tissues, with appropriate adjustments based on staining results), followed by a rapid rinse with distilled water. The sections were then quickly dehydrated through 70%, 80%, 90%, and absolute ethanol (10 s each), cleared in xylene three times (1–2 min each), and finally mounted with neutral resin for microscopic observation. Semi-quantitative assessment of staining intensity and area was performed using ImageJ software (version 1.54).

### 4.7. Cell Culture and Differentiation of Adipocytes

The 3T3-L1 cell line was obtained from the Cell Resource Center of Peking Union Medical College (National Biomedical Cell-Line Resource, Beijing, China). Cells were routinely cultured in MEM medium supplemented with 10% neonatal calf serum and 1% penicillin-streptomycin, following the protocol recommended by the American Type Culture Collection (ATCC, Manassas, VA, USA). Cultures were maintained at 37 °C with 5% CO_2_. All cell lines were authenticated by short tandem repeat (STR) analysis and routinely tested for mycoplasma contamination.

Adipogenic differentiation of 3T3-L1 cells was performed as previously described [79,80]. Briefly, cells were grown to 70–80% confluence and then incubated in DMEM/F12 (high glucose, Gibco, Grand Island, NY, USA) supplemented with 10% fetal bovine serum (FBS, Gibco), 0.125 mM indomethacin (Sigma-Aldrich), 1 nM triiodothyronine (T3, Sigma-Aldrich), 20 nM insulin (Sigma-Aldrich), 5 mM dexamethasone (Sigma-Aldrich), 1 μM rosiglitazone (MedChemExpress, Shanghai, China), and 0.5 mM 3-isobutyl-1-methylxanthine (IBMX, Sigma-Aldrich) for two days. Thereafter, cells were maintained in growth medium containing 1 nM T3 and 20 nM insulin for an additional four days, with the induction medium replaced every two days.

### 4.8. Oil Red O Staining

Oil Red O staining (C0158M, Beyotime, Shanghai, China) were carried out following the manufacturer’s instructions. Briefly, cells were covered with an appropriate volume of staining wash solution for 20 s, which was then aspirated. The cells were incubated with Oil Red O staining working solution for 10–20 min (the volume should be sufficient to evenly cover the cells). After removal of the staining working solution, the staining wash solution was added and allowed to stand for 30 s, then aspirated, followed by a 20-s wash with PBS. Finally, a suitable volume of PBS was added to cover the cells, and the samples were observed and photographed under a microscope.

### 4.9. Statistical Analysis

Data are presented as mean ± SEM. Normality was assessed using the Shapiro–Wilk and Kolmogorov–Smirnov tests. For comparisons between two groups, normally distributed data were analyzed using a two-tailed Student’s *t*-test, whereas non-normally distributed data were analyzed using the Mann–Whitney U test. For comparisons involving more than two groups, one-way ANOVA with appropriate post hoc tests was applied for normally distributed data, and the Kruskal–Wallis test followed by Dunn’s test was used for non-normally distributed data. Statistical analyses were performed using GraphPad Prism version 9.0.

## 5. Conclusions

In this study, we systematically compared four mouse models of pancreatic steatosis established based on distinct pathogenic mechanisms, revealing divergent temporal patterns, histological characteristics, and pathological outcomes. The long-term HFD model exhibited time-dependent progression of pancreatic steatosis, with onset occurring significantly later than hepatic steatosis, accompanied by progressive fibrosis, exocrine dysfunction, and macrophage infiltration. In contrast, the *ob/ob* obese model, despite displaying severe obesity, hepatic steatosis, and islet hyperplasia, did not develop substantial intrapancreatic fat deposition or mature adipocyte accumulation; however, pancreatic fibrosis and exocrine dysfunction were still evident, while macrophage infiltration remained unchanged, indicating that systemic obesity alone is insufficient to drive pancreatic steatosis. The caerulein combined with HFD model demonstrated a synergistic interaction between inflammation and metabolic stress, markedly accelerating intrapancreatic fat deposition, mature adipocyte accumulation, fibrosis, exocrine dysfunction, and macrophage infiltration compared with HFD alone. Finally, the focal model established by orthotopic injection of adipogenically differentiated 3T3-L1 cells provided a valuable tool for investigating the causal relationship between localized fat deposition and pancreatic pathology, effectively excluding systemic metabolic confounding factors, and was characterized by mature adipocyte deposition without significant fibrosis or exocrine dysfunction, accompanied by only mild local macrophage infiltration. Collectively, the establishment and systematic comparison of these four models provide a solid foundation for mechanistic studies, screening of potential intervention targets, and evaluation of therapeutic efficacy, while also offering important experimental evidence to support personalized prevention and treatment strategies for distinct subtypes of pancreatic steatosis in clinical settings.

## Data Availability

All data generated or analyzed during this study are included in this article. Further inquiries can be directed to the corresponding author.

## Supplementary Materials

The following supporting information can be downloaded at: https://www.mdpi.com/article/10.3390/ijms27104255/s1.

## Abbreviations

The following abbreviations are used in this manuscript:

HFD	High-fat diet
MRI	Magnetic resonance imaging
HE	Hematoxylin-eosin
IHC	Immunohistochemistry
ICD	International Classification of Diseases
CD	Chow diet
PBS	Phosphate-buffered saline
ATCC	American Type Culture Collection
PUMCH	Peking Union Medical College Hospital
STR	Short tandem repeat
FBS	Fetal bovine serum
T3	Triiodothyronine
IBMX	3-isobutyl-1-methylxanthine
PPARγ	Peroxisome proliferator-activated receptor gamma
SREBP-1c	Sterol regulatory element-binding protein 1c
NF-κB	Nuclear factor kappa-light-chain-enhancer of activated B cells
IL-6	Interleukin 6
TNF-α	Tumor necrosis factor alpha
