# Supplementary material for: Establishment and Comparative Analysis of Four Mouse Models for the Study of Pancreatic Steatosis
Source: Int J Mol Sci. 2026 May 10;27(10):4255. doi: 10.3390/ijms27104255 (PMC13207034; doi:10.3390/ijms27104255)
Supplement: Supplementary file 1 [file ijms-27-04255-s001.zip › ijms-4255883 Figure S1.pdf]

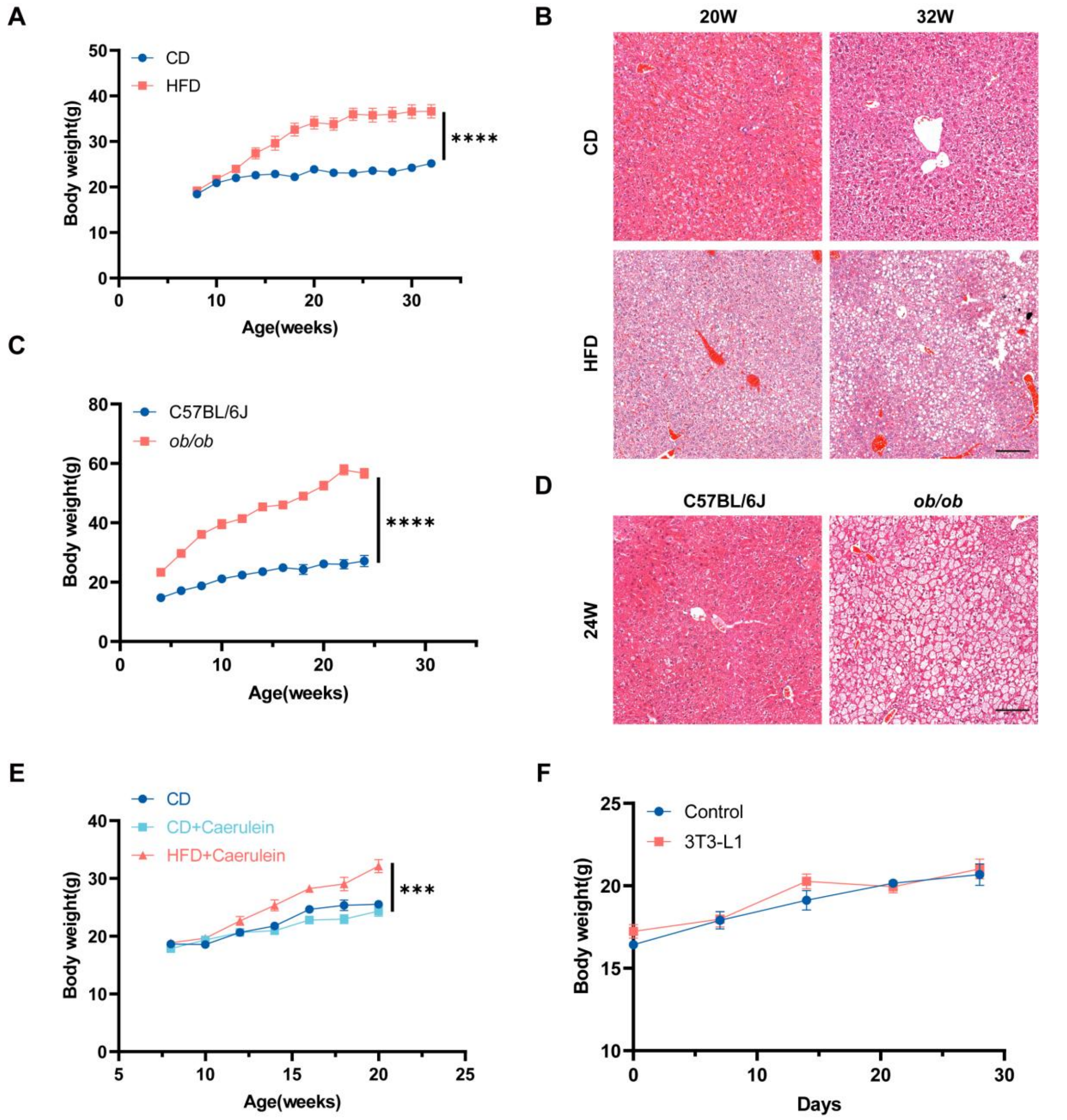

Figure S1. Body weight monitoring and hepatic histology in different mouse models. (**A**) Body weight changes in C57BL/6J mice fed a CD or HFD from 8 to 32 weeks of age (n = 8). (**B**) Representative HE staining of liver tissues from CD and HFD-fed C57BL/6J mice at 20 and 32 weeks of age. (**C**) Body weight changes in C57BL/6J and *ob/ob* mice from 8 to 24 weeks of age (n = 6). (**D**) Representative HE staining of liver tissues from C57BL/6J and *ob/ob* mice at 24 weeks of age. (**E**) Body weight changes in mice from the CD, CD+caerulein, and HFD+caerulein groups (n = 5). (**F**) Body weight changes in BABL/c nude mice following orthotopic injection of 3T3-L1 cells or control (n = 7). Scale bars = 200 μm. Data are presented as mean ± SEM. One-way ANOVA with Tukey's post hoc test (**E**), unpaired two-tailed Student's t-test (**A**, **C**, **F**). *** $p < 0.001$, **** $p < 0.0001$.
